# Clinical efficacy and drug resistance of ceftazidime-avibactam in the treatment of Carbapenem-resistant gram-negative bacilli infection

**DOI:** 10.3389/fmicb.2023.1198926

**Published:** 2023-08-17

**Authors:** Shuang Xiao, Qianwen Fu, Youhan Miao, Manna Zhao, Shengwei Lu, Jie Xu, Weifeng Zhao

**Affiliations:** ^1^Department of Infectious Diseases, The First Affiliated Hospital of Soochow University, Suzhou, China; ^2^Department of Critical Care Medicine, The First People’s Hospital of Tonglu, Hangzhou, China; ^3^Department of Infectious Diseases, The Third Affiliated Hospital of Nantong University, Nantong, China; ^4^Center of Clinical Laboratory, The First Affiliated Hospital of Soochow University, Suzhou, China

**Keywords:** ceftazidime-avibactam, Polymyxin B, carbapenem-resistant gram-negative bacilli, clinical efficacy, drug resistance

## Abstract

**Objective:**

To examine the clinical efficacy, safety, and resistance of Ceftazidime-Avibactam (CAZ-AVI) in patients with Carbapenem-resistant Gram-negative bacilli (CR-GNB) infections.

**Methods:**

We retrospectively analyzed relevant data of CR-GNB infected patients receiving CAZ-AVI treatment, analyzed relevant factors affecting drug efficacy, and compared the efficacy and safety with patients receiving Polymyxin B treatment.

**Results:**

A total of 139 patients were included. Agranulocytosis, septic shock, SOFA score, and CAZ-AVI treatment course were independent risk factors affecting the prognosis of patients with CR-GNB infection treated with CAZ-AVI while prolonging the treatment course of CAZ-AVI was the only protective factor for bacterial clearance. The fundamental indicators showed no statistically significant differences between CAZ-AVI and Polymyxin B treatment groups. At the same time, the proportion of patients treated with monotherapy was significantly higher in the CAZ-AVI group than in the Polymyxin B group (37.2% vs. 8.9%, *p* < 0.05), the 30-day mortality rate of the CAZ-AVI treatment group (27.7% vs. 46.7%, *p* = 0.027) was lower than that of the Polymyxin B treatment group. The 30-day clinical cure rate (59.6% vs. 40% *p* = 0.030) and 14-day microbiological clearance rate (42.6% vs. 24.4%, *p* = 0.038) were significantly higher in the CAZ-AVI than in the Polymyxin B treatment group. Eighty nine patients were monitored for CAZ-AVI resistance, and the total resistance rate was 14.6% (13/89). The resistance rates of Carbapenem-resistant *Klebsiella pneumoniae* (CRKP) and Carbapenem-resistant *Pseudomonas aeruginosa* (CRPA) to CAZ-AVI were 13.5 and 15.4%, respectively.

**Conclusion:**

CAZ-AVI has shown high clinical efficacy and bacterial clearance in treating CR-GNB infections. Compared with Polymyxin B, CAZ-AVI significantly improved the outcome of mechanical ventilation in patients with septic shock, agranulocytosis, Intensive Care Unit (ICU) patients, bloodstream infection, and patients with SOFA score > 6, and had a lower incidence of adverse events. We monitored the emergence of CAZ-AVI resistance and should strengthen the monitoring of drug susceptibility in clinical practice and the rational selection of antibiotic regimens to delay the onset of resistance.

## 1. Introduction

With the advent of broad-spectrum antibiotics, the spread of multidrug-resistant bacteria, represented by Gram-negative bacteria, has gradually become a significant global public health problem (Karakonstantis et al., 2020). Currently, carbapenems remain the first-line drugs for treating severe Gram-negative infections (Jee et al., 2018). However, with their increased use and intensity, the incidence and detection of Carbapenem-resistant *Enterobacterales* (CRE), Carbapenem-resistant *Pseudomonas aeruginosa* (CRPA), and Carbapenem-resistant *Acinetobacter baumannii* (CRAB) are on the rise globally, they also tend to carry Carbapenemases that inactivate most β-lactam antibiotics (Ehmann et al., 2013). Nevertheless, because of their limited antimicrobial action, adverse effects and resistance, as well as the emergence of extensively drug-resistant (XDR) bacteria and even fully pan-drug-resistant (PDR) bacteria have inspired us to continuously search for new anti-infective options.

Ceftazidime-Avibactam (CAZ-AVI), consisting of the third-generation Cephalosporin Ceftazidime (CAZ) and the novel β-lactamase inhibitor Avibactam (AVI), is effective against class A enzymes, class C enzymes (AmpC) and some class D enzymes [e.g., Oxacillinase (OXA)-48], while having no inhibitory activity against class B Metallo-β-lactamase-1 (NDM-1) (Endimiani et al., 2009). CAZ-AVI maintained good antibacterial activity against most *Enterobacterales* spp. Including AmpC, KPC and Extended-spectrum β-lactamase (ESBLs) producing *Enterobacterales*, but the antibacterial activity against *Acinetobacter baumannii* and *Pseudomonas aeruginosa* was dependent on their susceptibility to CAZ (Endimiani et al., 2009). CAZ-AVI was approved by the Food and Drug Administration (FDA) in February 2015 and by the National Medical Products Administration (NMPA) in May 2019 for the treatment of complicated urinary tract and complicated intra-abdominal infections caused by MDR or XDR gram-negative bacteria (Bonnefoy et al., 2004). The results of the systematic evaluation suggest that CAZ-AVI has positive pharmacological effects and can potentially be an empirical option for the treatment of severe Gram-negative bacterial infections. However, since most of the papers related to it are observational studies, case studies, or retrospective studies with smaller sample sizes, the clinical efficacy and safety of CAZ-AVI for treating CR-GNB infections also deserve further study.

To increase the evidence base for the use of CAZ-AVI, we retrospectively analyzed the clinical data of patients with CR-GNB infection, explored CAZ-AVI resistance and prognostic risk factors, and evaluated the efficacy and safety.

## 2. Materials and methods

### 2.1. Participants

#### 2.1.1. Basic clinical information of the patients

Clinical data, pathogenic data, and follow-up data were collected from patients with CR-GNB infection, including: 1. fundamental information of patients: gender, age, patient origin, and primary clinical diagnosis; 2. underlying diseases: diabetes, liver disease, cardiovascular disease, chronic kidney disease (CKD), chronic obstructive pulmonary diseases (COPD), personal history of malignancy; 3. Charlson comorbidity score (CCI), agranulocytosis, infectious shock, mechanical ventilation, Sepsis Related Organ Failure Assessment (SOFA) score, and continuous renal replacement therapy (CRRT); 4. infection status: initial site of infection (pulmonary infection, bloodstream infection, urinary tract infection, abdominal infection, central nervous system infection), and infection pathogens *(Klebsiella pneumoniae, Pseudomonas aeruginosa, Escherichia coli, Enterobacter cloacae)*; 5. medication: whether the drug was administered within 48 h, whether the drug was combined, the duration of anti-infective therapy, the results of bacterial *in vitro* drug susceptibility, and adverse reactions.

The primary outcome indexes of this study were the 30-day morbidity and mortality rate, and the secondary outcome index was the 30-day clinical cure rate, 14-day bacterial clearance rate, and the emergence of CAZ-AVI drug resistance.

#### 2.1.2. Study population and inclusion and exclusion criteria

We collected cases of CR-GNB infection treated with CAZ-AVI and Polymyxin B during hospitalization at the First Affiliated Hospital of Soochow University from July 1, 2019, to December 31, 2021. One hundred thirty-nine patients were eventually enrolled in this study, including 94 in the CAZ-AVI treatment group and 45 in the Polymyxin B treatment group.

**Figure 1:**
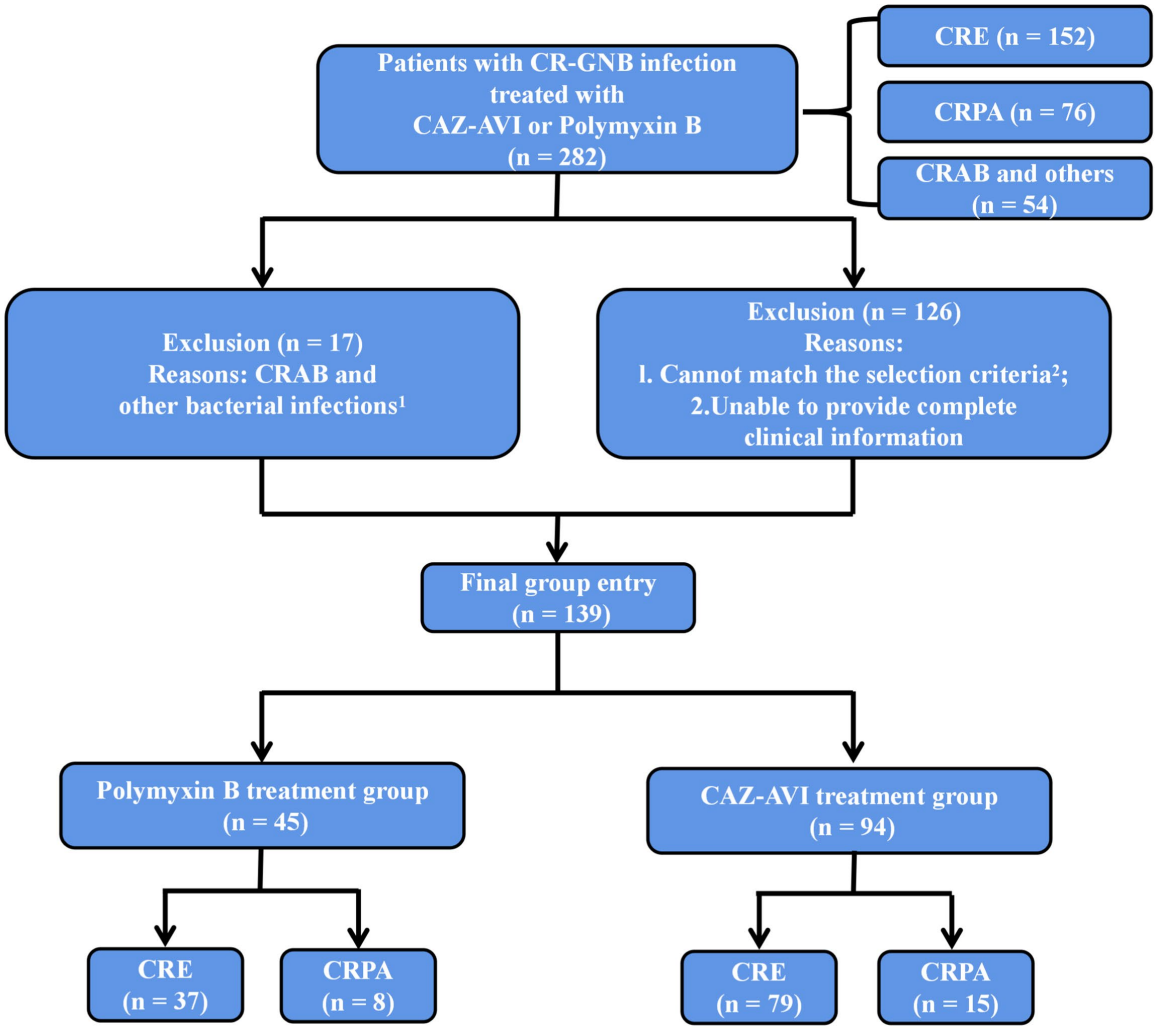
Inclusion and exclusion criteria. ^1^Treated with CAZ-AVI or Polymyxin B but not infected with CRE, CRPA or CRAB. ^2^Age < 18 years, treatment with CAZ-AVI or Polymyxin B for <72 h, CR-GNB colonization or contamination, CRAB (naturally resistant to CAZ-AVI) and other bacterial infections, multiple CR-GNB mixed infections or unspecified pathogens.

Inclusion Criteria: 1. age ≥ 18 years; 2. microbiological culture results confirming the definite presence of CR-GNB infection; 3. treatment with CAZ-AVI or Polymyxin B for ≥72 h (Figure 1).

Exclusion Criteria: 1. age < 18 years; 2. treatment with CAZ-AVI or Polymyxin B for <72 h; 3. CR-GNB colonization or contamination; 4. CRAB (naturally resistant to CAZ-AVI) and other bacterial infections; 5. multiple CR-GNB mixed infections or unspecified pathogens (Figure 1).

### 2.2. Isolation of strains and drug susceptibility testing

Bacterial isolation and culture identification refer to the National Clinical Laboratory Practice, using mass spectrometry identification, Vitek MS automatic rapid microbial mass spectrometry detection system, Vitek-2 Compact system AST-GN67 and AST-XN04 susceptibility cards for strain identification and drug susceptibility testing. Kirby-Bauer disk diffusion method was used to review the sensitivity of Imipenem, Meropenem, Piperacillin-Tazobactam, Amikacin and determine the sensitivity of Cefoperazone-Sulbactam, Ceftazidime-Avibactam (CAZ-AVI). Minimum inhibitory concentration (MIC) was determined for Polymyxin B by Broth-Micro Dilution method. The drug susceptibility results for Polymyxin were interpreted according to the European Committee on Antimicrobial Susceptibility Testing (EUCAST) standards and for the other drugs according to the American Clinical and Laboratory Standards Institute (CLSI) M100, 32nd ed. standards.

### 2.3. Statistical methods

1.Descriptive statistics were used to assess baseline characteristics of the entire cohort, CRE and *Pseudomonas aeruginosa* subpopulations, with discrete data expressed as counts and percentages and measures that conformed to a normal distribution and chi-squareness expressed as $\overset{-}{x}\pm SD$ using independent samples *t*-test. Data with skewed distribution were expressed as median (M) and interquartile ranges (IQR) using the nonparametric Mann–Whitney U test. The Pearson chi-square test or continuity-corrected chi-square test tested count data. The logistic multifactor regression analysis included some of the outcomes that were meaningful in the univariate analysis or other risk factors that might affect prognosis. The Receiver Operating Characteristic (ROC) curve was plotted and compared with the area under the curve, truncation value, susceptibility, and specificity, and the best value was calculated using the Jorden index. When the number of patients in the subgroup was too small for meaningful analysis, some variables were decomposed into single composite variables.

2.A controlled study of patients treated with CAZ-AVI and Polymyxin B was conducted to assess the efficacy and safety between the two groups by survival curves and COX regression analysis.

3.A descriptive analysis of the clinical data of CAZ-AVI-resistant patients was performed to explore the occurrence of drug resistance characteristics and mechanisms of drug resistance.

All statistical analyses were performed using IBM SPSS version 22.0 software (IBM Corporation, Armonk, NY, United States), and *p* values <0.05 were considered statistically significant.

## 3. Results

### 3.1. Participants

Among all 94 patients in the CAZ-AVI treatment group, the infectious organisms included 72 CRKP, two Carbapenem-resistant *Escherichia coli*, five Carbapenem-resistant *Enterobacter cloacae* (CREC), and 15 CRPA. More than 60% of the patients originated from the ICU. The most common types of infections were pulmonary infections and bloodstream infections. The underlying diseases of the patients included malignancy, diabetes mellitus, CKD in, cardiovascular disease, liver disease, and COPD. The median CCI score was 4 (IQR 2–6). The proportion of patients with infectious shock, granulocyte deficient patients and mechanically ventilated patients were all higher. The median SOFA score was 7 (IQR 5–9; Table 1).

**Table 1:** Clinical data of 94 patients treated with CAZ-AVI.

Clinical Features	CRE^1^ (*n* = 79)	CRPA (*n* = 15)	CR-GNB (*n* = 94)
Male [Cases (%)]	57 (72.2%)	11 (73.3%)	68 (72.3%)
Age (years, X ± s)	60.1 ± 18.8	55.6 ± 20.3	59.4 ± 19.0
*Patient origin [Cases (%)]*			
ICU ^2^	54 (68.4%)	8 (53.3%)	62 (66.0%)
General Ward	25 (31.6%)	7 (46.7%)	32 (34.0%)
*Comorbidities [Cases (%)]*			
Diabetes	21 (26.6%)	0 (0.0%)	21 (22.3%)
Liver Disease^3^	6 (7.6%)	1 (6.7%)	7 (7.0%)
CKD	13 (16.5%)	1 (6.7%)	14 (14.9%)
COPD	2 (2.5%)	0 (0.0%)	2 (2.1%)
Cardiovascular disease	6 (7.6%)	2 (13.3%)	8 (8.5%)
Personal history of malignancy^4^	29 (36.7%)	8 (53.3%)	37 (39.4%)
Charlson Comorbidity Index (M, IQR)	4 (2–6)	4 (2–6)	4 (2–6)
Infectious shock [Cases (%)]	32 (40.5%)	7 (46.7%)	39 (41.5%)
Agranulocytosis [Cases (%)]	26 (32.9%)	6 (40.0%)	32 (34.0%)
Mechanical Ventilation [Cases (%)]	36 (45.6%)	4 (26.7%)	40 (42.6%)
CRRT [Cases (%)]	13 (16.5%)	0 (0.0%)	13 (13.8%)
SOFA Score(IQR)	7 (5–10)	6 (4–8)	7 (5–9)
*Infection type [Cases (%)]*			
Pulmonary Infection	45 (57.0%)	9 (60.0%)	54 (57.4%)
Bloodstream infections	27 (34.2%)	4 (26.7%)	31 (33.0%)
Abdominal infections	3 (3.8%)	2 (13.3%)	5 (5.3%)
Urinary Tract Infections	3 (3.8%)	0 (0.0%)	3 (3.2%)
Central nervous system infections	1 (1.3%)	0 (0.0%)	1 (1.1%)
CAZ-AVI resistance (Drug Resistance/Testing)	7/60 (11.7%)	0/5 (0.0%)	7/65 (10.8%)

ICU, intensive care unit; CKD, chronic kidney disease; COPD, chronic obstructive pulmonary disease; CCI, Charlson comorbidity index; CRRT, continuous renal replacement therapy; SOFA, systemic infection-associated Sexual Organ Failure Score; CAZ-AVI, Ceftazidime-Avibactam; CRE, Carbapenem-resistant Enterobacteria; CRPA, Carbapenem-resistant *Pseudomonas aeruginosa*; CR-GNB, Carbapenem-resistant gram-negative bacilli.

1 Including 72 cases of CRKP, 2 cases of Carbapenem-resistant *Escherichia coli*, and 5 cases of Carbapenem-resistant *Enterobacter cloacae*.

2 ICU patient sources included infection ICU (*n* = 13), respiratory ICU (*n* = 20), critical care ICU (*n* = 18), surgical ICU (*n* = 5), hematology ICU (*n* = 4), and cardiovascular surgery ICU (*n* = 2).

3 Including post-hepatitis B cirrhosis (*n* = 3), post-liver transplantation (*n* = 1), acute drug-related liver failure (*n* = 1), and schistosomiasis liver disease (*n* = 2).

4 Including acute myeloid leukemia (*n* = 22), acute lymphoblastic leukemia (*n* = 4), MDS (*n* = 1), lymphoma (*n* = 2), colon cancer (*n* = 2), esophageal cancer (*n* = 1), pancreatic cancer (*n* = 1), gastric malignancy (*n* = 1), prostate malignancy (*n* = 1), pituitary tumor (*n* = 1), primary liver cancer (*n* = 1).

### 3.2. Drug susceptibility results

As shown in Table 2, the 79 clinical isolates of CRE strains had a high resistance rate to Penicillin antibiotics, Cephalosporins, Fluoroquinolones, and Two Carbapenems (Imipenem and Meropenem) (≥ 96.2%). Resistance rates to aminoglycosides (Amikacin and Gentamicin) were also high (≥ 79.7%). But the rate of resistance to Tigecycline was relatively low (43, 54.4%). Sixty of the seventy-nine patients with CRE infection were tested for CAZ-AVI, 7 (11.7%) CAZ-AVI-resistant strains were detected, 10 cases were tested for Polymyxin B, and 2 cases were tested for Fosfomycin. All of them were susceptible strains (Table 2). CRPA isolates showed high resistance to Penicillin antibiotics (Ticarcillin: 15, 100%, Piperacillin: 12, 80%), Penicillin antibiotics + β-lactamase inhibitors (Ticarcillin/Clavulanic acid: 13, 86.7%), Cephalosporins + β-lactamase inhibitors (Cefoperazone/Sulbactam: 11, 73.3%). However, good susceptibility was maintained to Aminoglycosides and Fluoroquinolones. Five cases of CRPA were tested for CAZ-AVI drug susceptibility, and no CAZ-AVI-resistant strains were found (Table 2).

**Table 2:** *In vitro* drug susceptibility results of the isolated CR-GNB against common clinical antibiotics.

Antibacterial drugs	CRE [*n* = 79, *n* (%)]			CRPA [*n* = 15, *n* (%)]		
	Drug-resistant	Moderate	susceptible	Drug-resistant	Moderate	susceptible
Ticarcillin	79 (100%)	0 (0.0%)	0 (0.0%)	15 (100.0%)	0 (0.0%)	0 (0.0%)
Piperacillin	79 (100%)	0 (0.0%)	0 (0.0%)	12 (80.0%)	3 (20.0%)	0 (0.0%)
Ticarcillin/Clavulanic acid	79 (100%)	0 (0.0%)	0 (0.0%)	13 (86.7%)	2 (13.3%)	0 (0.0%)
Piperacillin/Tazobactam	77 (97.5%)	1 (1.3%)	1 (1.3%)	13 (86.7%)	1 (6.7%)	1 (6.7%)
Cefoperazone/Sulbactam	73 (92.4%)	2 (2.5%)	4 (5.1%)	11 (73.3%)	4 (26.7%)	0 (0.0%)
Ceftriaxone	79 (100%)	0 (0.0%)	0 (0.0%)	-	-	-
Ceftazidime	79 (100%)	0 (0.0%)	0 (0.0%)	9 (60.0%)	4 (26.7%)	2 (13.3%)
Aztreonam	75 (94.9%)	0 (0.0%)	4 (5.1%)	13 (86.7%)	2 (13.3%)	0 (0.0%)
Meropenem	76 (96.2%)	0 (0.0%)	3 (3.8%)	12 (80.0%)	3 (20.0%)	0 (0.0%)
Imipenem	76 (96.2%)	1 (1.3%)	2 (2.5%)	14 (93.3%)	0 (0.0%)	1 (6.7%)
Gentamicin	64 (81.0%)	2 (2.5%)	13 (16.5%)	3 (20.0%)	1 (6.7%)	11 (73.3%)
Amikacin	63 (79.7%)	1 (1.3%)	15 (19.0%)	3 (20.0%)	0 (0.0%)	12 (80.0%)
Levofloxacin	77 (97.5%)	1 (1.3%)	1 (1.3%)	5 (33.3%)	5 (33.3%)	5 (33.3%)
Ciprofloxacin	76 (96.2%)	1 (1.3%)	2 (2.5%)	4 (26.7%)	4 (26.7%)	7 (46.7%)
Tigecycline	43 (54.4%)	16 (20.3%)	20 (25.3%)	15 (100.0%)	0 (0.0%)	0 (0.0%)
Ceftazidime-Avibactam ^1^	7 (11.7%)	0 (0.0%)	53 (88.3%)	0 (0.0%)	0 (0.0%)	5 (100.0%)
Polymyxin B ^2^	0 (0.0%)	0 (0.0%)	10 (100.0%)	-	-	-
Fosfomycin ^3^	0 (0.0%)	0 (0.0%)	2 (100.0%)	-	-	-

1 CAZ-AVI drug susceptibility tests were performed in 60 of the CRE cases and 5 of the CRPA cases.

2 Polymyxin B susceptibility testing was performed in 10 of the CRE cases.

3 Fosfomycin susceptibility testing was performed in 2 of the 3CRE cases.

### 3.3. Efficacy of CAZ-AVI

Of the 94 patients with CR-GNB infection, 49 received CAZ-AVI therapy within 48 h, including 43 in the CRE group and 6 in the CRPA group. Twenty-nine patients in the CRE group received CAZ-AVI monotherapy, and 50 patients received CAZ-AVI-based combination therapy with the paramount combination. The main combination regimens included Tigecycline, Aztreonam, Carbapenems, Cephalosporins and Tigecycline + Aztreonam. The median duration of CAZ-AVI treatment was 9 days (IQR, 6–12). Nine cases in the CRPA group received CAZ-AVI combination therapy. The most common combination regimen was combined with Aztreonam, followed by Carbapenems, Tigecycline + Aztreonam and Fluoroquinolones. The median duration of CAZ-AVI treatment was 7 days (IQR, 6–10). The results showed a 30-day clinical cure rate of 59.6%, a 14-day bacterial clearance rate of 42.6%, and a 30-day morbidity and mortality rate of 27.7% for CAZ-AVI in the treatment of CR-GNB. 30-day morbidity and mortality rate, 14-day bacterial clearance rate and 30-day clinical cure rate were not significantly different between CRE and CRPA groups (Table 3).

**Table 3:** Antimicrobial-related parameters and outcome indicators of CAZ-AVI treatment.

Treatment Features	CRE (*n* = 79)	CRPA (*n* = 15)	CR-GNB (*n* = 94)	*p-*value
Use CAZ-AVI in 48H [cases (%)]	43 (54.4%)	6 (40.0%)	49 (52.1%)	0.305
Monotherapy [cases (%)]	29 (36.7%)	6 (40.0%)	35 (37.2%)	0.809
CAZ-AVI combination drug regimen [cases (%)]	50 (63.3%)	9 (60.0%)	59 (62.8%)	
Fluoroquinolones	1 (2.0%)	1 (11.1%)	2 (2.1%)	
Aminoglycosides	1 (2.0%)	1 (11.1%)	2 (2.1%)	
Cephalosporins	5 (10.0%)	0 (0.0%)	5 (5.3%)	
Fosfomycin	2 (4.0%)	0 (0.0%)	2 (2.1%)	
Tigecycline	15 (30.0%)	0 (0.0%)	15 (16.0%)	
Aztreonam	12 (24.0%)	4 (44.4%)	16 (17.0%)	
Carbapenems	12 (24.0%)	2 (22.2%)	14 (14.9%)	
Tigecycline + Aztreonam	2 (4.0%)	1 (11.1%)	3 (3.2%)	
CAZ-AVI treatment course (IQR)	9 (6–12)	7 (6–10)	8 (6–12)	0.253
30-day clinical cure rate	44 (55.7%)	12 (80%)	56 (59.6%)	0.079
14-day bacterial clearance rate	34 (36.2%)	6 (40.0%)	40 (42.6%)	0.827
30-day morbidity and mortality rate	23 (29.1%)	3 (20%%)	26 (27.7%)	0.469

### 3.4. Prognostic risk factor analysis

The 30-day morbidity and mortality rate and 14-day bacterial clearance rate were used as clinical indicators to evaluate the prognosis and drug efficacy of CAZ-AVI for CR-GNB patients, respectively. The results of the univariate analysis based on 30-day death showed that ICU patients, infectious shock, agranulocytosis and mechanical ventilation were risk factors for poor prognosis of CR-GNB infection treated with CAZ-AVI, and the rate of patient morbidity and mortality was significantly higher with increased SOFA score and a short course of CAZ-AVI application (Table 4). The risk factors that were statistically different in the univariate analysis (agranulocytosis, mechanical ventilation, infectious shock, SOFA score, ICU patients, and duration of CAZ-AVI application) were included in the multivariate logistic regression analysis, which showed that agranulocytosis, infectious shock, high SOFA score, and short duration of CAZ-AVI application were the risk factors for poor prognosis in patients with CR-GNB infection (Table 5). The analysis of factors that may influence the 14-day bacterial clearance rate (included factors were gender, age, ICU patients, CCI, infectious shock, agranulocytosis, mechanical ventilation, CRRT, SOFA score, type of infection, infectious agent, combination therapy, CAZ-AVI application within 48 h and CAZ-AVI course) showed that the results of the univariate analysis showed that age, ICU patients, infectious shock, mechanical ventilation, SOFA score and duration of CAZ-AVI application were independent influencing factors for bacterial clearance at 14 days in patients with CR-GNB infection. Further significant results from the univariate analysis were included in the multifactorial logistic regression analysis, which showed that the long course of CAZ-AVI application was the only protective factor for bacterial clearance (Table 6).

**Table 4:** Analysis of risk factors for poor prognosis (30-day morbidity and mortality).

Risk factors	Death within 30 days	No death within 30 days	*p-*value
	(*n* = 26)	(*n* = 68)	
Male [cases (%)]	18 (69.2%)	50 (73.5%)	0.681
Age (years, X ± s)	64.2 ± 16.6	57.6 ± 19.7	0.137
ICU patients [cases (%)]	22 (84.6%)	40 (58.8%)	0.018^#^
Charlson Comorbidity Index (IQR)	5 (3–7)	3 (2–6)	0.169
Infectious shock [cases (%)]	20 (76.9%)	19 (27.9%)	< 0.05^#^
Agranulocytosis [cases (%)]	14 (53.8%)	18 (26.4%)	0.027^#^
Mechanical Ventilation [cases (%)]	16 (61.5%)	24 (35.3%)	0.021^#^
CRRT [cases (%)]	4 (15.4%)	9 (13.2%)	0.787
SOFA Score (IQR)	10 (7–14)	6 (4–8)	< 0.05^#^
Lung infection [cases (%)]	14 (53.8%)	40 (58.8%)	0.666
Bloodstream infection [cases (%)]	7 (26.9%)	24 (35.3%)	0.445
Abdominal infection [cases (%)]	4 (15.4%)	1 (1.5%)	0.099
CRKP infection	22 (84.6%)	50 (73.5%)	0.256
CRPA infection	2 (7.7%)	13 (19.1%)	0.176
Use CAZ-AVI within 48H [cases (%)]	12 (46.2%)	37 (54.4%)	0.479
Co-medication [cases (%)]	17 (65.4%)	42 (61.8%)	0.749
CAZ-AVI treatment (IQR)	7 (5–9)	9 (7–12)	0.005

#Represented the *p*-value < 0.05, meaning that there was the significant difference between the data.

**Table 5:** Multifactorial analysis of factors affecting 30-day morbidity and mortality in patients with CR-GNB infection treated by CAZ-AVI.

Risk factors	*b* value	*Sb* value	wald c2	*p*-value	*OR*	95% confidence interval of EXP (B)	
						lower-bound	upper-bound
Agranulocytosis	1.355	0.632	4.594	0.032	3.878	1.123	13.393
Mechanical Ventilation	0.604	0.728	0.688	0.407	1.829	0.439	7.612
Infectious shock	1.63	0.675	5.832	0.016	5.105	1.36	19.167
SOFA Score	−0.202	0.100	4.060	0.044	0.817	0.672	0.995
ICU patients	0.956	0.881	1.176	0.278	2.601	0.462	14.63
CAZ-AVI procedure	0.222	0.088	6.281	0.012	1.248	1.050	1.485

**Table 6:** Univariate and multifactorial analysis of 14-day bacterial clearance in patients with CR-GNB infection treated with CAZ-AVI.

Influence factors	Single factor analysis		Multi-factor analysis	
	*OR* (95% CI)	*p* value	*OR* (95% CI)	*p* value
Male	0.632 (0.247–1.615)	0.338		
Age	0.968 (0.945–0.009)	0.007^#^	0.972 (0.945–1.000)	0.051
ICU patients	2.853 (1.184–6.879)	0.02^#^	1.401 (0.417–4.702)	0.585
Charlson Comorbidity index	0.881 (0.740–1.049)	0.154		
Infectious shock	2.839 (1.183–6.815)	0.019^#^	1.961 (0.652–5.896)	0.231
Agranulocytosis	1.373 (0.573–3.286)	0.477		
Mechanical ventilation	2.513 (1.062–5.947)	0.036^#^	1.351 (0.405–4.515)	0.625
CRRT	0.844 (0.260–2.737)	0.777		
SOFA score	0.874 (0.775–0.985)	0.027^#^	0.959 (0.819–1.123)	0.603
Lung infection	1.700 (0.741–3.899)	0.210		
Bloodstream infection	0.577 (0.242–1.375)	0.215		
Abdominal infection	1.118 (0.178–7.022)	0.906		
CRKP infection	2.119 (0.816–5.497)	0.123		
CRPA infection	0.820 (0.271–2.484)	0.725		
Use CAZ-AVI within 48H	0.974 (0.430–2.209)	0.950		
Co-medication	1.182 (0.505–2.763)	0.700		
CAZ-AVI treatment	1.121 (1.108–1.235)	0.020^#^	1.147 (1.03–1.278)	0.013^#^

#Represented the *p*-value < 0.05, meaning that there was the significant difference between the data.

### 3.5. Emergence of CAZ-AVI drug resistance

We monitored the occurrence of CAZ-AVI resistance while treating severe Gram-negative bacterial infections with CAZ-AVI and Polymyxin B. We monitored CAZ-AVI resistance in 89 patients with CR-GNB disease in the CAZ-AVI treatment group and Polymyxin B treatment group. The overall resistance rate was 14.6%, and CRKP and CRPA to CAZ-AVI resistance rates were 13.5 and 15.4%, respectively. Only two cases of Carbapenem-resistant *Enterobacter cloacae* were tested for drug susceptibility, and the resistance rate was 50% (Table 7).

**Table 7:** Subgroup analysis table based on 30-day morbidity and mortality rate.

Infectious pathogens	susceptible	Drug-resistant
CRKP	64/74 (86.5%)	10/74 (13.5%)
CRPA	11/13 (84.6%)	2/13 (15.4%)
CREC	1/2 (50.0%)	1/2 (50.0%)
Total	76/89 (85.4%)	13/89 (14.6%)

### 3.6. Comparison of clinical efficacy and safety of CAZ-AVI and PolymyxinB

#### 3.6.1. Basic clinical information

The proportion of single drugs in the CAZ-AVI treatment group was much more significant than that in the Polymyxin B treatment group. In contrast, the proportion of their combination was much lower than that in the Polymyxin B treatment group. At the same time, there was no statistically significant difference in whether the corresponding antimicrobial therapy was initiated within 48 h and the duration of drug use. The comparison of efficacy between the two groups showed that the CAZ-AVI group had a significantly lower 30-day morbidity and mortality rate than the Polymyxin B-treated group. In contrast, the 30-day clinical cure rate and 14-day bacterial clearance rate were significantly higher than those of the control group. The incidence of acute kidney injury was not significantly different between the two groups, while the incidence of diarrhea and skin pigmentation was statistically significant (Table 8).

**Table 8:** Basic clinical information, efficacy, and adverse effects of patients in CAZ-AVI treatment group and Polymyxin B treatment group.

Clinical Features	CAZ-AVI treatment group	Polymyxin B treatment group	*p* value
	*N* = 94	*N* = 45	
Male [Cases (%)]	68 (72.3%)	34 (75.6%)	0.688
Age (years, X ± s)	59.4 ± 19.0	53.7 ± 16.1	0.085
*Patient source [Cases (%)]*			
ICU	62 (66.0%)	22 (48.9%)	0.054
General Ward	32 (34.0%)	23 (51.1%)	0.054
*Comorbidities [Cases (%)]*			
Diabetes	21 (22.3%)	12 (26.7%)	0.575
Liver Diseases	7 (7.4%)	5 (11.1%)	0.472
CKD	14 (14.9%)	6 (13.3%)	0.806
COPD	2 (2.1%)	0 (0.0%)	0.324
Cardiovascular disease	8 (8.5%)	1 (2.2%)	0.159
Personal history of malignancy	37 (39.4%)	24 (53.2%)	0.120
Charlson Comorbidity Index(M, IQR)	4 (2–6)	3 (2–4)	0.296
Infectious shock [Cases (%)]	39 (41.5%)	19 (42.2%)	0.935
Agranulocytosis [Cases (%)]	32 (34.0%)	17 (37.8%)	0.666
Mechanical Ventilation [Cases (%)]	40 (42.6%)	18 (40.0%)	0.775
CRRT [Cases (%)]	13 (13.8%)	2 (4.4%)	0.169
SOFA Score (IQR)	7 (5–9)	6 (4–8)	0.109
*Infection type [Cases (%)]*			
Pulmonary Infection	54 (57.4%)	25 (55.6%)	0.833
Bloodstream infection	31 (33.0%)	15 (33.3%)	0.967
Abdominal infections	5 (5.3%)	3 (6.7%)	0.750
Urinary Tract Infections	3 (3.2%)	1 (2.2%)	0.749
Central nervous system infections	1 (1.1%)	1 (2.2%)	0.592
*Pathogenic microorganisms [cases (%)]*			
CRKP	72 (76.6%)	32 (71.1%)	0.486
CRPA	15 (16.0%)	8 (15.6%)	0.787
*Escherichia coli*	2 (2.1%)	3 (6.7%)	0.115
*Enterobacter cloacae*	5 (5.3%)	2 (4.4%)	0.428
Initiate treatment within 48H	49 (52.1%)	22 (48.9%)	0.721
*Treatment options [cases (%)]*			
Single drug	35 (37.2%)	4 (8.9%)	*p* < 0.05^#^
Co-medication	59 (62.8%)	41 (91.1%)	*p* < 0.05^#^
Fluoroquinolones	2 (2.1%)	6 (13.3%)	
Aminoglycosides	2 (2.1%)	3 (6.7%)	
Cephalosporins	5 (5.3%)	3 (6.7%)	
Fosfomycin	2 (2.1%)	0 (0.0%)	
Tigecycline	15 (16.0%)	10 (22.2%)	
Aztreonam	16 (17.0%)	2 (4.4%)	
Carbapenems	14 (14.9%)	17 (37.8%)	
Tigecycline + Aztreonam	3 (3.2%)	0 (0.0%)	
Application Duration Days (IQR)	8 (6–12)	7 (5–11)	0.125
14-day bacterial clearance rate [cases (%)]	40 (42.6%)	11 (24.4%)	0.038^#^
30-day clinical cure rate [cases (%)]	56 (59.6%)	18 (40.0%)	0.030^#^
30-day morbidity and mortality rate [cases (%)]	26 (27.7%)	21 (46.7%)	0.027^#^
*Adverse reactions [cases (%)]*			
Diarrhea	3 (3.2%)	0 (0.0%)	*p* < 0.05^#^
Renal insufficiency	4 (4.3%)	4 (8.9%)	1.00
Skin pigmentation	0 (0.0%)	12 (26.7%)	*p* < 0.05^#^
*Drug resistance testing (resistance/testing)*			
CAZ-AVI	7/65 (10.8%)	6/24 (25.0%)	
Polymyxin B	0/10 (0.0%)	3/14 (21.4%)	

#### 3.6.2. Survival curve

**Figure 2:**
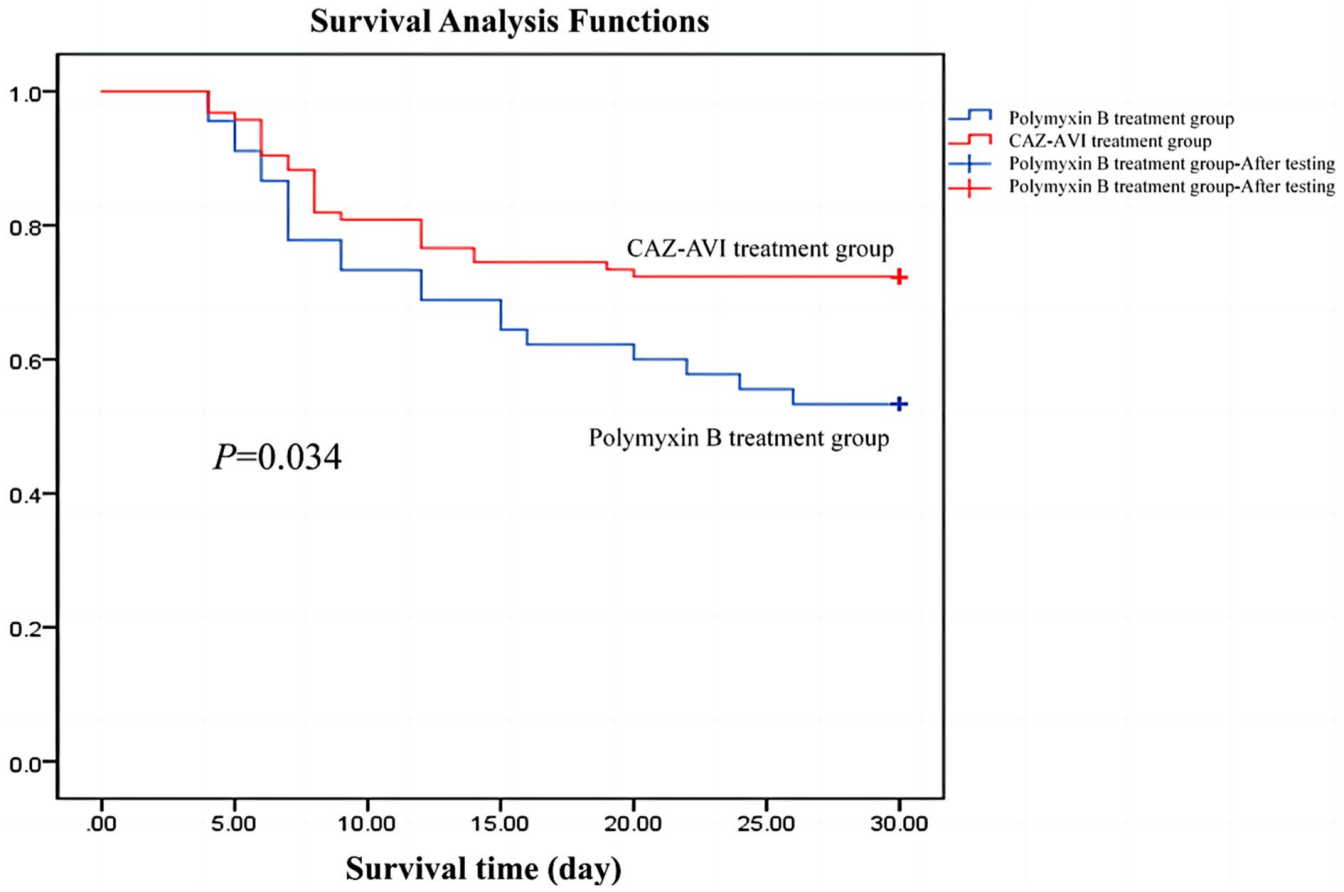
Survival curves of CAZ-AVI treatment group and Polymyxin B treatment group.

We followed both groups for 30 days and plotted Kaplan–Meier survival curves, which showed that patients in the CAZ-AVI treatment group had higher 30-day survival rates than those in the Polymyxin B group (Figure 2).

#### 3.6.3. Results of subgroup analysis of 30-day morbidity and mortality rates

Subgroup analysis of 30-day mortality rates for outcome indicators (included groups were infectious shock, mechanical ventilation, granulocyte deficiency, ICU-derived patients, pulmonary infection, bloodstream infection, CRKP infection, CCI, and SOFA score) showed that CAZ-AVI significantly reduced the 30-day mortality rate in the infected shock group, the mechanical ventilation group, the agranulocytosis group, the ICU patient group, the bloodstream infection group, and the SOFA score > 6 compared with the Polymyxin B group. In contrast, CAZ-AVI did not show better efficacy than Polymyxin B in the pulmonary infection group, the CRKP infection group, and the CCI > 3 group (Table 9).

**Table 9:** Subgroup analysis table based on 30-day morbidity and mortality rate.

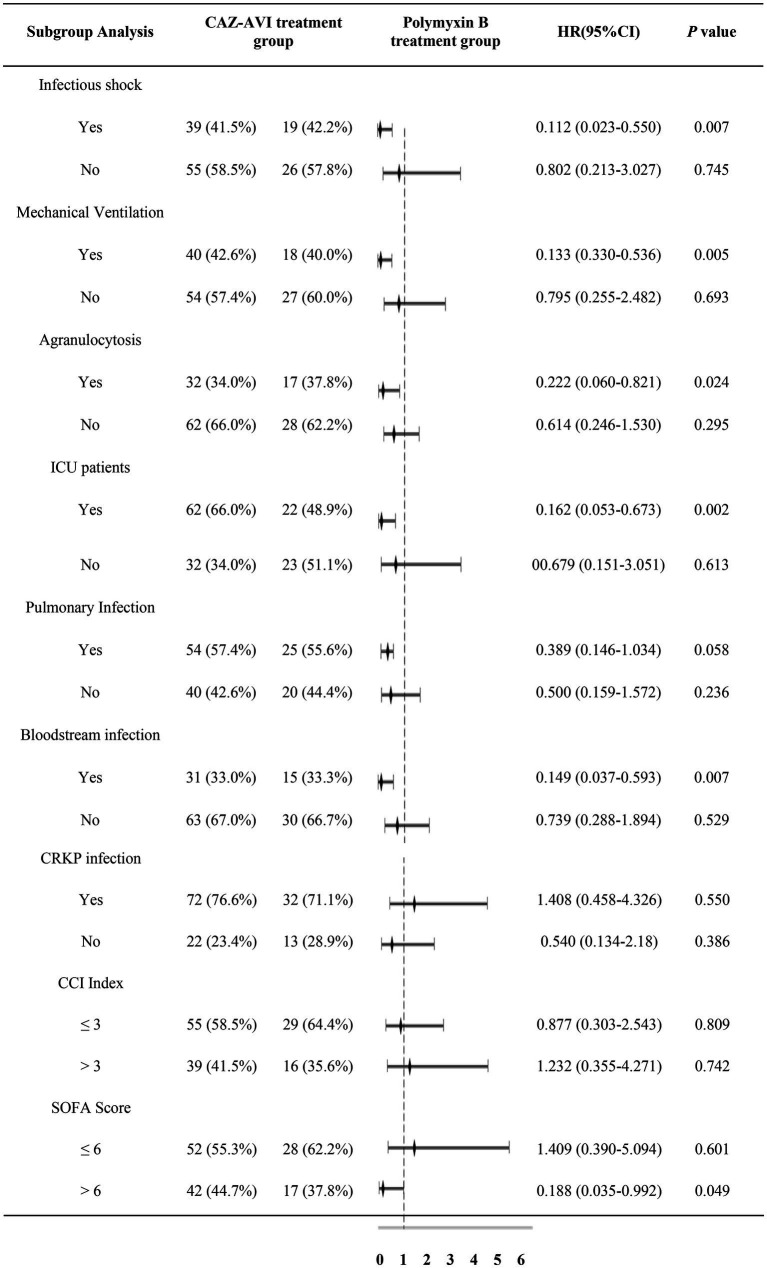

#### 3.6.4. Emergence of drug resistance

In both the CAZ-AVI treatment group and the Polymyxin B treatment group, some patients were monitored for resistance to both drugs, with a 14.6% probability of resistance to CAZ-AVI and a 12.5% probability of resistance to Polymyxin B. However, we cannot provide accurate statistical evidence due to incomplete data on drug resistance monitoring. Further studies are still needed to monitor the emergence of drug resistance for both drugs.

## 4. Discussion

With the widespread use of Carbapenem antibacterial drugs in the past decade, the incidence and detection rate of Carbapenem-resistant bacteria has been increasing year by year, and the morbidity and mortality rate of infections caused by these resistant pathogens can be as high as 50% due to the lack of effective treatment options (Senchyna et al., 2019). *In vitro* tests demonstrated that CAZ-AVI has good antibacterial activity against *Enterobacterales* spp. and *Pseudomonas aeruginosa*. In this study, 94 patients with CR-GNB infection received CAZ-AVI-based anti-infective regimens, which showed a 30-day morbidity and mortality rate of 27.7%, a 14-day bacterial clearance rate of 42.6%, and a 30-day clinical cure rate of 59.6%. Jorgensen et al. (2019) performed a descriptive analysis of patients treated with CAZ-AVI-based therapy for multidrug-resistant Gram-negative bacteria (MDR-GNB) infection in 55.6% of ICU patients with a median CCI of 4 and a median SOFA score of 5. Their results showed a 30-day morbidity and mortality rate and a clinical cure rate of 17.2 and 70.9%, respectively. The higher morbidity and mortality rates and significantly lower clinical cure rates of the outcome indicators in this study were considered to be related to the different degrees of the criticality of the included patients, who had higher SOFA scores and a higher proportion of patients with bloodstream infections, infectious shock, and mechanical ventilation. Despite the higher criticality of the included patients, CAZ-AVI showed better efficacy.

A total of 15 patients infected with CRPA were included in this study, with a 30-day morbidity and mortality rate of 20% (3/15) and a 30-day clinical cure and a 14-day bacterial clearance rate of 80 and 40%. Compared to CRE, there needs to be more clinical experience with CAZ-AVI for treating patients with CRPA infection, and fewer drugs are available for treating CRPA (Sader et al., 2015). However, our findings suggest that CAZ-AVI has better efficacy than other antibiotics, further demonstrating the feasibility of CAZ-AVI for treating CR-GNB. CAZ-AVI appears to be a new alternative drug. Vena et al. (2020) retrospectively analyzed 41 patients with MDR gram-negative bacterial infections other than CRE treated with CAZ-AVI, 33 of which were CRPA infections. They showed a clinical cure rate of 87.8%, similar to the present study. A study by Santevecchi et al. (2018) also showed good efficacy of CAZ-AVI in patients with *Pseudomonas aeruginosa* infections. Our study is similar to existing studies showing that CAZ-AVI can be used as a clinical treatment option for patients infected with CRPA. The latest guidelines from the Infectious Diseases Society of America (IDSA) have identified CAZ-AVI as a first-line agent for the treatment of refractory *P. aeruginosa* infections. Established studies on the timing of CAZ-AVI treatment have found CAZ-AVI to be more effective in the early treatment of severe CR-GNB infections and in salvage therapy. A study by Falcone et al. (2020) found that in Carbapenemase-producing *Klebsiella pneumoniae* (KPC-producing *Klebsiella pneumoniae*, KPC-KP) bloodstream infections patients, the median time from culture to initiation of appropriate antibiotic therapy was significantly shorter in surviving patients than in patients who died (8.5 h vs. 48 h, *p* = 0.014), and receiving appropriate antibiotic therapy within 24 h significantly reduced the 30-day morbidity and mortality rate in patients (29.1% vs. 63.8%). Jorgensen et al. (2019) had similar findings, and receiving CAZ-AVI treatment was an independent prognostic factor for clinical success in patients with CR-GNB infection. In addition, CAZ-AVI treatment may still be effective in patients with CRE infection who have failed early treatment with other drugs. A retrospective study showed that CAZ-AVI-based salvage therapy regimens significantly reduced the 30-day mortality rate in patients with KPC-KP bloodstream infections (Tumbarello et al., 2019). Guimarães et al. (2019) analyzed the efficacy of CAZ-AVI salvage therapy in 29 patients with Polymyxin-resistant KPC-KP infections, all strains susceptible to CAZ-AVI. The results showed an excellent clinical cure rate of 82.7% and mortality rates of 31.0 and 51.7% at 14 and 30 day, respectively.

Several factors influence the drug efficacy of CAZ-AVI. Our study showed that infectious shock, granulocyte deficiency and SOFA score > 6 were risk factors for poor prognosis with CAZ-AVI treatment. At the same time, prolonged use of CAZ-AVI was its protective factor, with results similar to the above study. Tumbarello et al. (2021) retrospectively divided CAZ-AVI for treating KPC-producing *Klebsiella pneumoniae* infections, all patients were treated with CAZ-AVI alone or with ≥1 other effective antimicrobial agent, and the 30-day morbidity and mortality rate was 25%. Analysis of risk factors associated with morbidity and mortality showed that agranulocytosis, infectious shock, lower respiratory tract infections, and drug dose adjustment due to renal insufficiency were risk factors for CAZ-AVI drug efficacy. In contrast, prolonged CAZ-AVI infusion time was the only protective factor. Another study showed similar findings: analysis of risk factors for drug efficacy showed that infectious shock, granulocyte deficiency, CCI > 3 and mechanical ventilation were independent risk factors for patient death while receiving CAZ-AVI treatment was the only protective factor for survival (Tumbarello et al., 2019). The results of the existing studies mentioned above are similar to our findings.

In this study, a retrospective analysis of patients treated with CAZ-AVI and Polymyxin B-based monotherapy or combination for CR-GNB infection showed that the 14-day bacterial clearance rate and 30-day clinical cure rate in the CAZ-AVI group were 42.6 and 59.6%, respectively, which were significantly better than those in the Polymyxin B group, while the 30-day morbidity and mortality rate was 27.7%, which was significantly lower than that in the Polymyxin B group. Based on the 30-day morbidity and mortality rate based on the outcome index, we performed subgroup analysis. We showed that CAZ-AVI significantly improved the prognosis of patients with infectious shock, mechanical ventilation, agranulocytosis, ICU patients, bloodstream infection, and SOFA score > 6. In a meta-analysis of 281 cases with CRE infections, the results showed that treatment with CAZ-AVI significantly improved clinical cure rates and reduced morbidity and mortality compared with other susceptible antibiotic regimens; subgroup analysis of infection types showed that CAZ-AVI significantly improved clinical outcomes in patients with bloodstream infections and improved microbiological clearance in patients with complicated urinary tract infections (Zhong et al., 2018). CAZ-AVI is mostly (80–90%) excreted *via* the kidneys as a prototype, resulting in higher drug concentrations in the urinary tract. Therefore, it is more suitable for patients with urinary tract infections. However, it was not analyzed separately as a subgroup because only four patients with urinary tract infections were included in this study. The data sample size was too small, and the data were heavily biased. Tsolaki et al. (2020) compared 41 patients receiving a CAZ-AVI-containing based regimen and 36 patients with severe CRE infection treated with other susceptible antibiotic regimens, all of whom received mechanical ventilation and had SOFA scores >3 and APACHE II scores >10. The results showed that the CAZ-AVI-based regimen significantly improved 28-day survival, bacterial clearance, and clinical cure rates in critically ill patients.

There is still great controversy about CAZ-AVI monotherapy or combination therapy. In this study, more than 60% of patients were treated with a combination regimen of CAZ-AVI, mainly in combination with Tigecycline, Aminoglycosides, Carbapenems, Fosfomycin and Aztreonam. There was no statistically significant difference in the 30-day morbidity and mortality rate between patients treated with CAZ-AVI monotherapy and combination therapy (9/35, 25.7% vs. 17/59, 28.8%, *p* = 0.815). Previous studies have shown that CAZ-AVI effectively treats CRE infections with monotherapy and combination therapy. However, some combination regimens, such as Aminoglycosides, Polymyxins, Fluoroquinolones, and Tigecycline, have not significantly improved therapeutic efficacy (Onorato et al., 2019). An experimental study analyzed three clinical isolates of KPC-3 producing *Klebsiella pneumoniae*, all with mutations in ompk35 and ompk36, susceptible to CAZ-AVI and Polymyxin B. The results showed that combining Polymyxin B with CAZ-AVI had no *in vitro* bactericidal effect on KPC-producing *Klebsiella pneumonia* compared to CAZ-AVI alone. There was no improvement in either *in vitro* activity or *in vivo* bactericidal efficacy of KPC-producing *Klebsiella pneumonia*, and it did not improve survival. This combination should be avoided in favor of either the drug alone or another combination that may be more effective and less toxic (Borjan et al., 2020). Tigecycline inhibits protein synthesis by reversibly binding to bacterial ribosomal subunits for antibacterial purposes (WHO Informal Working Group, 2003), but *in vitro* drug susceptibility tests showed that CAZ-AVI in combination with Tigecycline was synergistic for only 5% of CRKP (Ojdana et al., 2019). Fosfomycin inhibits bacterial cell wall synthesis and increases the uptake of other antibiotics. The combination of CAZ-AVI and Fosfomycin reduced the MIC of multidrug-resistant *Pseudomonas aeruginosa in vitro* assays (Kazmierczak et al., 2018). Carbapenems are also often used in combination with CAZ-AVI. Gaibani et al. evaluated the antimicrobial efficacy of CAZ-AVI in combination with six commonly used antimicrobial drugs (Ertapenem, Imipenem, Meropenem, Gentamicin, Tigecycline, and Ciprofloxacin) against KPC-producing *Klebsiella pneumoniae* agents in an *in vitro* trial, showing that only the combination of CAZ-AVI and Imipenem or Meropenem had synergistic activity (Gaibani et al., 2017). The latest IDSA guidelines on treating drug-resistant Gram-negative infections recommend routine combination therapy for Carbapenem-resistant *Enterobacterales* infections. This may be due to AVI’s protective effect against hydrolysis by Carbapenemases. Conversely, Carbapenems are anti-selective for the blaKPC-3 mutation, which can lead to CAZ-AVI resistance (Mac Vane et al., 2017). It is worth mentioning that in patients with Metallo-beta-lactamase-producing CRE infections, the treatment regimen of CAZ-AVI combined with Aztreonam has shown promising efficacy. Falcone et al. (2021) conducted a prospective study in patients with Metallo-beta-lactamase-producing gram-negative bacteria bloodstream infections. They showed that the 30-day morbidity and mortality rate and the incidence of acute kidney injury in patients receiving the combination of CAZ-AVI and Aztreonam were significantly lower than those in the control group. Recent studies suggest that although combination therapy is not superior to monotherapy in terms of clinical efficacy, it may reduce the recurrence rate and delay the development of drug resistance. A total of three cases of CAZ-AVI resistance were monitored in our study, all of which occurred in the CAZ-AVI treatment group. The results of the risk factor analysis showed that CAZ-AVI monotherapy did not affect the drug efficacy of CAZ-AVI compared with combination therapy, which is consistent with the results of previous studies. However, the following biases may exist in our study: first, in actual clinical practice, there is a clinical tendency to administer CAZ-AVI-containing combination therapy to patients with severe disease and serious underlying diseases such as immunodeficiency; second, the complex and diverse treatment regimens in the combination therapy group and the small sample size of the subgroup make the conclusions somewhat limited.

Both Polymyxins and CAZ-AVI maintain good antibacterial activity against CR-GNB infections. Polymyxins mediate bacterial death by altering the permeability of bacterial cell membranes. In recent years, multidrug-resistant infections have become increasingly tricky, and Polymyxins are once again one of the salvage treatment options. However, in our study, the CAZ-AVI group had a significantly lower 30-day mortality rate (27.7% vs. 46.7%, *p* = 0.027) and a significantly higher 30-day clinical cure rate (59.6% vs. 40%, *p* = 0.030) and 14-day microbial clearance rate (42.6% vs. 24.4%, *p* = 0.038) than the Polymyxin B treatment group. In terms of adverse effects, the incidence of systemic skin pigmentation was significantly higher in the Polymyxin B group than in the CAZ-AVI group (26.7% vs. 0%, *p* < 0.05), with a statistically significant difference, and the incidence of acute kidney injury was not significantly different between the two groups (4/45, 8.9% vs. 4/94, 4.3%). A meta-analysis found that the 30-day mortality rate of patients with CRE infection treated with CAZ-AVI was 39.5%, and the clinical cure rate was 68.4% compared with Carbapenems and Polymyxins, suggesting that the use of CAZ-AVI can effectively control CRE infection and reduce the mortality rate (Temkin et al., 2017). Qureshi et al. (2012) found that the overall morbidity and mortality rate in patients with CRKP-infected bacteremia was 39%, with patients receiving Polymyxin monotherapy patients having a high morbidity and mortality rate of 57.1%. In comparison, the combination therapy group had a morbidity and mortality rate of 12.5%, a statistically significant difference, suggesting that combination therapy may be an effective way to improve survival in patients with CRKP bacteremia. In conclusion, the clinical efficacy of CAZ-AVI in the treatment of CR-GNB was superior to that of Polymyxin B, and the incidence of adverse effects was significantly lower. In our study, the Polymyxin B resistance rate was 12.5% (3/24), which was relatively high. However, the sample size was small because only 24 patients were tested for Polymyxin drug susceptibility. The data were biased, therefore, regarding the resistance rate, we still need to conduct further studies based on the expansion of the sample size.

Among 94 patients with CR-GNB infection treated with CAZ-AVI, the emergence of resistant strains was detected in seven cases (7/65, 10.8%), including two bloodstream infections and one pulmonary infection, both of which were CRKP. Unfortunately, genetic testing for Carbapenemases was performed in only one case and confirmed that the development of resistance was associated with Metallo-beta-lactamases production. During treatment with CAZ-AVI, 89 patients were tested for CAZ-AVI resistance; the overall resistance rate was 14.6% (9/64). The resistance rates of CRKP and CRPA to CAZ-AVI were 13.5 and 15.4%, respectively. Although CAZ-AVI resistance is still uncommon, it is increasingly reported. Once resistance occurs, the clinical dilemma of having no drug available may be faced. Secondly, the morbidity and mortality rate of patients infected with resistant strains of CAZ-AVI seems to be high (nearly 40%) (Winkler et al., 2015). Internationally relevant bacterial surveillance data show that most *Enterobacteriaceae* have a low rate of resistance to CAZ-AVI (< 2.6%) (Wise et al., 2018). In contrast, *Pseudomonas aeruginosa* has a relatively high resistance rate, reaching 4–8% (Nichols et al., 2016). The current study suggests that the emergence of resistance is not related to the presence or absence of previous CAZ-AVI treatment. In 2015, Humphries et al. (2015) reported the first CAZ-AVI-resistant strain (MIC, 32/4ug/ml) isolated from a patient without previous CAZ-AVI treatment. They considered its resistance associated with KPC enzyme production. Gaibani et al. (2020) also reported three KPC-KP infections that were not treated with CAZ-AVI but developed drug resistance. Therefore, monitoring changes in drug susceptibility, both before and during drug administration, is particularly important. There are few clinically available alternative treatment options for CAZ-AVI resistance. A recent study reported a maximum percentage of resistant strains of up to 12.7% in patients treated with CAZ-AVI-based monotherapy, highlighting the possible role of combination therapy in proper clinical management (Haidar et al., 2017). In a prospective observational study, patients with Metallo-beta-lactamase-producing CRE bloodstream infections treated with CAZ-AVI in combination with Aztreonam had a significantly lower 30-day morbidity and mortality rate than the other susceptible antibiotic treatment groups (Falcone et al., 2021).

There are some limitations in this study: 1. the number of sample cases is small, especially some subgroup data, and a large-scale multi-surgical randomized controlled study is needed; 2. this paper only included patients with single strain infection and single site infection, lacking clinical analysis of patients with CRE mixed infection; 3. the results of this research can only serve as a limited recommendation for the clinical selection of antibiotics because part of the strains could not be completely tested for CAZ-AVI and polymyxin B medication susceptibility *in vitro*; 4. due to laboratory technical limitations, most strains were not tested for Carbapenemase genes, and the effects of different genotypes of Carbapenemase on drug efficacy could not be explored; 5. the susceptibility testing with automated systems might be unreliable; 6. the changes in strains on CAZ-AVI MIC values during treatment were not monitored, and the timing of drug resistance could not be accurately described. The monitored drug-resistant strains of CAZ-AVI were not further explored for resistance mechanisms.

## 5. Conclusion

CAZ-AVI demonstrated high clinical efficacy and bacterial clearance in treating CR-GNB infections, potentially improving patient survival and providing a better option for treating CR-GNB infections. CAZ-AVI significantly improved mechanical ventilation in patients with septic shock, granulocytopenia, ICU patients, bloodstream infections, and SOFA score > 6 when compared to Polymyxin B, with a lower incidence of adverse effects. Simultaneously, we tracked the emergence of CAZ-AVI resistance. We should improve drug susceptibility monitoring in clinical practice in order to select appropriate antibiotic regimens and delay the development of drug resistance.

## Data availability statement

The original contributions presented in the study are included in the article/supplementary material, further inquiries can be directed to the corresponding authors.

## Author contributions

WZ conceived the work. QF collected the data. SX and QF analyzed the data and drafted the article. YM, JX, MZ, SL, and WZ commented and edited the article. All authors have read and agreed to the published version of the manuscript.

## Funding

This study was supported by the Pfizer Global Medical Grants (76080151).

## Conflict of interest

The authors declare that the research was conducted in the absence of any commercial or financial relationships that could be construed as a potential conflict of interest.

## Publisher’s note

All claims expressed in this article are solely those of the authors and do not necessarily represent those of their affiliated organizations, or those of the publisher, the editors and the reviewers. Any product that may be evaluated in this article, or claim that may be made by its manufacturer, is not guaranteed or endorsed by the publisher.

## References

[ref1] WHO Informal Working Group (2003). International classification of ultrasound images in cystic echinococcosis for application in clinical and field epidemiological settings. Acta Trop. 85, 253–261. doi: 10.1016/S0001-706X(02)00223-1, PMID: 12606104

[ref2] Bonnefoy A. Dupuis-Hamelin C. Steier V. Delachaume C. Seys C. Stachyra T.. (2004). *In vitro* activity of AVE1330A, an innovative broad-spectrum non-beta-lactam beta-lactamase inhibitor. J. Antimicrob. Ch. 54, 410–417. doi: 10.1093/jac/dkh358, PMID: 15254025

[ref3] Borjan J. Meyer K. Shields R. K. Wenzler E. (2020). Activity of ceftazidime-avibactam alone and in combination with Polymyxin B against Carbapenem-resistant *Klebsiella pneumoniae* in a tandem *in vitro* time-kill/*in vivo* galleria mellonella survival model analysis. Int. J. Antimicrob. Agents 55:105852. doi: 10.1016/j.ijantimicag.2019.11.009 31770627

[ref4] Ehmann D. Jahić H. Ross P. Gu R. F. Hu J. Durand-Réville T. F.. (2013). Kinetics of avibactam inhibition against class A, C, and D β-lactamases. J. Biol. Chem. 288, 27960–27971. doi: 10.1074/jbc.M113.485979, PMID: 23913691 PMC3784710

[ref5] Endimiani A. Choudhary Y. Bonomo R. (2009). In vitro activity of NXL104 in combination with beta-lactams against *Klebsiella pneumoniae* isolates producing KPC Carbapenemases. Antimicrob. Agents. Ch. 53, 3599–3601. doi: 10.1128/AAC.00641-09 PMC2715587 19528274

[ref6] Falcone M. Bassetti M. Tiseo G. Giordano C. Nencini E. Russo A.. (2020). Time to appropriate antibiotic therapy is a predictor of outcome in patients with bloodstream infection caused by KPC-producing *Klebsiella pneumoniae*. Crit. Care 24:29. doi: 10.1186/s13054-020-2742-9 32000834 PMC6993311

[ref7] Falcone M. Daikos G. L. Tiseo G. Bassoulis D. Giordano C. Galfo V.. (2021). Efficacy of ceftazidime-avibactam plus Aztreonam in patients with bloodstream infections caused by Metallo-β-lactamase-producing *Enterobacterales*. Clin. Infect. Dis. 72, 1871–1878. doi: 10.1093/cid/ciaa586, PMID: 32427286

[ref8] Gaibani P. Lewis R. E. Volpe S. L. Giannella M. Campoli C. Landini M. P.. (2017). In vitro interaction of ceftazidime-avibactam in combination with different antimicrobials against KPC-producing *Klebsiella pneumoniae* clinical isolates. Int. J. Infect. Dis. 65, 1–3. doi: 10.1016/j.ijid.2017.09.017, PMID: 28951106

[ref9] Gaibani P. Re M. C. Campoli C. Viale P. L. Ambretti S. (2020). Bloodstream infection caused by KPC-producing *Klebsiella pneumoniae* resistant to ceftazidime/avibactam: epidemiology and genomic characterization. Clin. Microbiol. Infec. 26, 516.e1–516.e4. doi: 10.1016/j.cmi.2019.11.011, PMID: 31740422

[ref10] Guimarães T. Nouér S. A. Martins R. C. R. Neto L. V. P. Martins W. M. B. S.. (2019). *Enterobacteriales* ceftazidime-avibactam as salvage therapy for infections caused by Coresistant to Carbapenems and Polymyxins. Antimicrob. Agents. Ch. 63:e00528–19. doi: 10.1128/AAC.00528-19, PMID: 31358592 PMC6761521

[ref11] Haidar G. Clancy C. J. Shields R. K. Hao B. Cheng S. Nguyen M. H. (2017). Mutations in blaKPC-3 that confer ceftazidime-avibactam resistance encode novel KPC-3 variants that function as extended-Spectrum β-lactamases. Antimicrob. Agents. Ch. 61:e02534–16. doi: 10.1128/AAC.02534-16, PMID: 28223379 PMC5404534

[ref12] Humphries R. M. Yang S. Hemarajata P. Ward K. W. Hindler J. A.. (2015). First report of ceftazidime-avibactam resistance in a KPC-3-expressing *Klebsiella pneumoniae* isolate. Antimicrob. Agents. Ch. 59, 6605–6607. doi: 10.1128/AAC.01165-15, PMID: 26195508 PMC4576121

[ref13] Jee Y. Carlson J. Rafai E. Musonda K. Huong T. T. G. Daza P.. (2018). Antimicrobial resistance: a threat to global health. Lancet Infect. Dis. 18, 939–940. doi: 10.1016/S1473-3099(18)30471-7 30152350

[ref14] Jorgensen S. C. J. Trinh T. D. Zasowski E. J. Lagnf A. M. Bhatia S.. (2019). Real-world experience with ceftazidime-avibactam for multidrug-resistant gram-negative bacterial infections. Open. Forum. Infect. Di. 6:ofz522. doi: 10.1093/ofid/ofz522, PMID: 31890725 PMC6934163

[ref15] Karakonstantis S. Kritsotakis E. Gikas A. (2020). Pandrug-resistant gram-negative bacteria: a systematic review of current epidemiology, prognosis and treatment options. J. Antimicrob. Chemother. 75, 271–282. doi: 10.1093/jac/dkz401 31586417

[ref16] Kazmierczak K. M. de Jonge B. L. M. Stone G. G. Sahm D. F. (2018). In vitro activity of ceftazidime/avibactam against isolates of *Enterobacteriaceae* collected in European countries: INFORM global surveillance 2012-15. J. Anrimicrob. Chemoth. 73, 2782–2788. doi: 10.1093/jac/dky266 30010894

[ref17] Mac Vane S. H. Pandey R. Steed L. L. Kreiswirth B. N. Chen L. (2017). Emergence of Ceftolozane-Tazobactam-resistant *Pseudomonas aeruginosa* during treatment is mediated by a single amp C structural mutation. Antimicrob. Agents. Ch. 61:e01183–17. doi: 10.1128/AAC.01183-17, PMID: 28947473 PMC5700322

[ref18] Nichols W. W. de Jonge B. L. Kazmierczak K. M. Karlowsky J. A. Sahm D. F. (2016). In vitro susceptibility of global surveillance isolates of *Pseudomonas aeruginosa* to ceftazidime-avibactam (INFORM 2012 to 2014). Antimicrob. Agents. Ch. 60, 4743–4749. doi: 10.1128/AAC.00220-16 PMC4958170 27216074

[ref19] Ojdana D. Gutowska A. Sacha P. Majewski P. Wieczorek P. Tryniszewska E. (2019). Activity of ceftazidime-avibactam alone and in combination with Ertapenem, Fosfomycin, and Tigecycline against Carbapenemase-producing *Klebsiella pneumoniae*. Microb. Drug Resist. 25, 1357–1364. doi: 10.1089/mdr.2018.0234, PMID: 31295055

[ref20] Onorato L. Di Caprio G. Signoriello S. Coppola N. (2019). Efficacy of ceftazidime/avibactam in monotherapy or combination therapy against Carbapenem-resistant gram-negative bacteria: a meta-analysis. Int. J. Antimicrob. Agents 54, 735–740. doi: 10.1016/j.ijantimicag.2019.08.025 31479738

[ref21] Qureshi Z. A. Paterson D. L. Potoski B. A. Kilayko M. C. Sandovsky G. Sordillo E.. (2012). Treatment outcome of bacteremia due to KPC-producing *Klebsiella pneumoniae*: superiority of combination antimicrobial regimens. Antimicrob. Agents. Ch. 56, 2108–2113. doi: 10.1128/AAC.06268-11, PMID: 22252816 PMC3318350

[ref22] Sader H. Castanheira M. Flamm R. Mendes R. E. Farrell D. J. Jones R. N. (2015). Ceftazidime/avibactam tested against gram-negative bacteria from intensive care unit (ICU) and non-ICU patients, including those with ventilator-associated pneumonia. Int. J. Antimicrob. Agents 46, 53–59. doi: 10.1016/j.ijantimicag.2015.02.022, PMID: 25956844

[ref23] Santevecchi B. A. Smith T. T. Mac Vane S. H. (2018). Clinical experience with ceftazidime/avibactam for treatment of antibiotic-resistant organisms other than *Klebsiella pneumoniae*. Int. J. Antimicrob. Agents 51, 629–635. doi: 10.1016/j.ijantimicag.2018.01.016, PMID: 29408227

[ref24] Senchyna F. Gaur R. Sandlund J. Truong C. Tremintin G. Kültz D.. (2019). Diversity of resistance mechanisms in Carbapenem-resistant *Enterobacteriaceae* at a health care system in northern California, from 2013 to 2016. Diagn. Microbiol. Infect. Dis. 93, 250–257. doi: 10.1016/j.diagmicrobio.2018.10.004, PMID: 30482638 PMC7155946

[ref26] Temkin E. Torre-Cisneros J. Beovic B. Benito N. Giannella M. Gilarranz R.. (2017). Ceftazidime-avibactam as salvage therapy for infections caused by Carbapenem-resistant organisms. Antimicrob. Agents. Ch. 61:e01964–16. doi: 10.1128/AAC.01964-16, PMID: 27895014 PMC5278727

[ref27] Tsolaki V. Mantzarlis K. Mpakalis A. Malli E. Tsimpoukas F. Papagiannitsis C.. (2020). Ceftazidime-avibactam to treat life-threatening infections by Carbapenem-resistant pathogens in critically ill mechanically ventilated patients. Antimicrob. Agents. Ch. 64:e02320–19. doi: 10.1128/AAC.02320-19, PMID: 31818820 PMC7038311

[ref28] Tumbarello M. Raffaelli F. Giannella M. Mantengoli E. Mularoni A. Venditti M.. (2021). Ceftazidime-avibactam use for *Klebsiella pneumoniae* Carbapenemase-Producing *K. pneumoniae* infections: a retrospective observational multicenter study. Clin. Infect. Dis. 73, 1664–1676. doi: 10.1093/cid/ciab176, PMID: 33618353

[ref29] Tumbarello M. Trecarichi E. Corona A. De Rosa F. G. Bassetti M.. (2019). Efficacy of ceftazidime-avibactam salvage therapy in patients with infections caused by *Klebsiella pneumoniae* Carbapenemase-producing *K. pneumoniae*. Clin. Infect. Dis. 68, 355–364. doi: 10.1093/cid/ciy492 29893802

[ref30] Vena A. Giacobbe D. Castaldo N. Cattelan A. Mussini A.. (2020). Clinical experience with ceftazidime-avibactam for the treatment of infections due to multidrug-resistant gram-negative Bacteria other than Carbapenem-resistant. Antibiotics 9:71. doi: 10.3390/antibiotics9020071, PMID: 32050434 PMC7168189

[ref31] Winkler M. L. Papp-Wallace K. M. Hujer A. M. Domitrovic T. N. Hujer K. M. Hurless K. N.. (2015). Unexpected challenges in treating multidrug-resistant gram-negative bacteria: resistance to ceftazidime-avibactam in archived isolates of *Pseudomonas aeruginosa*. Antimicrob. Agents. Ch. 59, 1020–1029. doi: 10.1128/AAC.04238-14, PMID: 25451057 PMC4335889

[ref32] Wise M. G. Estabrook M. A. Sahm D. F. Stone G. G. Kazmierczak K. M. (2018). Prevalence of mcr-type genes among colistin-resistant *Enterobacteriaceae* collected in 2014-2016 as part of the INFORM global surveillance program. PLoS One 13:e0195281. doi: 10.1371/journal.pone.0195281, PMID: 29608599 PMC5880376

[ref33] Zhong H. Zhao X. Zhang Z. Gu Z. Zhang C. Gao Y.. (2018). M. Evaluation of the efficacy and safety of ceftazidime/avibactam in the treatment of gram-negative bacterial infections: a systematic review and meta-analysis. Int. J. Antimicrob. Agents 52, 443–450. doi: 10.1016/j.ijantimicag.2018.07.004 30012440

